# Development of sensitizer peptide-fused endolysin Lys1S-L9P acting against multidrug-resistant gram-negative bacteria

**DOI:** 10.3389/fmicb.2023.1296796

**Published:** 2023-11-23

**Authors:** Su Min Son, Joonbeom Kim, Sangryeol Ryu

**Affiliations:** ^1^Department of Food and Animal Biotechnology, Research Institute of Agriculture and Life Sciences, Seoul National University, Seoul, Republic of Korea; ^2^Department of Agricultural Biotechnology, Seoul National University, Seoul, Republic of Korea; ^3^Center for Food and Bioconvergence, Seoul National University, Seoul, Republic of Korea

**Keywords:** endolysin, sensitizer peptide, fusion endolysin, antimicrobial agent, multidrug resistance, gram-negative bacteria

## Abstract

The advent of multidrug-resistant (MDR) bacteria poses a major threat to public health, garnering attention to novel antibiotic replacements. Endolysin, a bacteriophage-derived cell wall-degrading enzyme, is a promising alternative to conventional antibiotics. However, it is challenging to control Gram-negative bacteria due to the presence of the outer membrane that shields the peptidoglycan layer from enzymatic degradation. To overcome this threshold, we constructed the fusion endolysin Lys1S-L9P by combining endolysin LysSPN1S with KL-L9P, a sensitizer peptide known to extend efficacy of antibiotics by perturbing the outer membrane of Gram-negative bacteria. In addition, we established a new endolysin purification procedure that increases solubility allowing a 4-fold increase in production yield of Lys1S-L9P. The sensitizer peptide-fused endolysin Lys1S-L9P exhibited high bactericidal effects against many MDR Gram-negative pathogens and was more effective in eradicating biofilms compared to LysSPN1S. Moreover, Lys1S-L9P showed potential for clinical use, maintaining stability at various storage temperatures without cytotoxicity against human cells. In the *in vivo Galleria mellonella* model, Lys1S-L9P demonstrated potent antibacterial activity against MDR Gram-negative bacteria without inducing any toxic activity. This study suggest that Lys1S-L9P could be a potential biocontrol agent to combat MDR Gram-negative bacteria.

## Introduction

With the advent of antibiotics, various powerful drugs have been used for more than half a century to counter infectious diseases caused by pathogenic bacteria. However, the overuse and misuse of antibiotics have led humankind to enter a new era where antibiotic-resistant strains have become prevalent (English and Gaur, 2010). The increasing emergence of multidrug-resistant (MDR) Gram-negative bacteria compromises our ability to effectively cure infections, posing a major threat to human health. According to the Centers for Disease Control and Prevention in 2019, more than 2.8 million antimicrobial-resistant infections have been reported to occur in the U.S. each year, causing more than 35,000 deaths. The World Health Organization (WHO) published a list of antibiotic-resistant “priority pathogens” for which new antibiotics are urgently needed, and emphasized the threat of MDR Gram-negative bacteria, especially on the most critical group including *Acinetobacter baumannii*, *Pseudomonas aeruginosa*, and *Enterobacteriaceae*. These MDR Gram-negative pathogens are a recognized public health threat in hospitals or nursing homes, causing severe infections with high mortality rates (Ghaith et al., 2017; Lim et al., 2019; Zhou et al., 2019; Mahmoud et al., 2020; Heidari et al., 2022; Radera et al., 2022; Saha et al., 2022). Therefore, it is paramount to develop novel antimicrobial agents to combat various MDR Gram-negative bacteria that does not cause drug-resistance.

Endolysins are phage-encoded enzymes that play a crucial role in the end of the phage life cycle, resulting in the release of the bacteriophage. These enzymes degrade the peptidoglycan layer of the bacterial cell wall, leading to a loss in osmotic balance within the cytoplasm, resulting in bacteriolysis (Young et al., 2000; Fischetti, 2005; Loessner, 2005; Oliveira et al., 2012). Endolysins are spotlighted as promising antimicrobial agents that can be alternatives to antibiotics due to several characteristics. First, exogenously applied endolysins are able to cause rapid lysis of the host bacteria, which is attributed to their specific action on the peptidoglycan layer (Borysowski et al., 2006). Their mode of action is less likely to induce bacterial resistance compared to conventional antibiotics because the peptidoglycan structure remains relatively constant across bacteria strains, reducing the chance of resistance development (Liu et al., 2023). Moreover, endolysins efficiently destabilize and eradicate bacteria embedded in the biofilm matrix, which exhibit more resistance (Shen et al., 2013; Lood et al., 2015; Schuch et al., 2017; Cha et al., 2019). For these reasons, numerous studies have focused on developing endolysins with therapeutic potential to control the MDR pathogens, especially, Gram-positive bacteria (Kim and Seo, 2023; Lu et al., 2023).

Despite such advantages, it is challenging to use endolysins to control Gram-negative bacteria because the outer membrane prohibits endolysins from accessing the peptidoglycan layer. The outer membrane comprises phospholipids, integral membrane proteins, lipoproteins, and glycolipids, called lipopolysaccharide (LPS) (Lugtenberg and Van Alphen, 1983). The hydrophobicity of the outer membrane act as a hurdle for large hydrophilic molecules like endolysins to permeate the cell wall of Gram-negative bacteria (Chen, 2007). In addition, LPS molecules, composed of lipophilic lipid A, an oligosaccharide core, and a long polysaccharide chain, can provide the structural integrity of the phospholipids, further limiting the endolysin to pass through the outer membrane. To overcome these thresholds, many researchers proposed several strategies to target Gram-negative bacteria using different protein engineering approaches, such as artilysins, lysocins, and innolysins (Defraine et al., 2016; Heselpoth et al., 2019; Zampara et al., 2021). However, although the reported engineered endolysins did succeed in controlling Gram-negative bacteria, some problems still need to be solved, such as the low yield of the proteins, narrow host ranges, and low efficiency in the *in vivo* model (Gerstmans et al., 2020; Zampara et al., 2020; Chen et al., 2021). Therefore, there is of urgent need for research on developing novel fusion endolysins to address these challenges.

Sensitizer peptides are compounds that sensitize the outer membrane of Gram-negative bacteria, which can be expected to extend the use of antibiotics for Gram-positive antibiotics to Gram-negative bacteria. The mode of action of sensitizer peptides is associated with the relocation of anionic LPS molecules in the membrane to positions where impermeability against antimicrobial agents is lost. Compared to conventional cell penetrating peptides, sensitizer peptides do not demolish or penetrate the membrane, but can demix membranes and potentiate the activity of antimicrobial agents such as antibiotics or endolysins. KL-L9P is a pro-hinged α-helical amphipathic peptide with sensitizer properties. It can promote rearrangement of the bacterial membrane rather than destroying it, allowing most Gram-positive antibiotics to pass through the outer membrane. It also displayed no hemolytic activities and did not abolish bacterial and eukaryotic membranes while still retaining weak bactericidal activity against Gram-negative bacteria (Hyun et al., 2020). Based on these properties, we hypothesized that endolysins and the sensitizer peptide KL-L9P could be engineered to enable efficient passage through the impermeable outer membrane.

In this study, we constructed the fusion endolysin, Lys1S-L9P, by fusing the sensitizer peptide KL-L9P onto the endolysin LysSPN1S and established a procedure to refine the fusion endolysin at a higher yield. It exhibited enhanced antibacterial activity and biofilm removal efficacy against MDR Gram-negative bacteria than the native endolysin LysSPN1S with a prolonged shelf life and no cytotoxicity to human cells. Furthermore, the therapeutic potential of the Lys1S-L9P was also verified in an *in vivo* model, where the fusion endolysin rescued larvae from bacterial infections causing significant reductions in bacterial loads. This study provides evidence that the fusion of endolysin with sensitizer peptides can be successfully applied as a therapeutic antimicrobial agent to control a wide range of MDR Gram-negative bacteria.

## Materials and methods

### Bacterial strains and growth conditions

Bacterial strains used in this study are listed in Supplementary Table S3. All strains were cultured in Luria-Bertani (LB broth, BD Difco) medium with aeration at 37°C. When necessary, antibiotics or chemicals were added to LB medium or agar at the following concentrations: kanamycin (Km, 50 μg/mL) and isopropyl β-D-thiogalactopyranoside (IPTG, 0.5 mM).

### Molecular gene cloning

The endolysin LysSPN1S gene was amplified from the genomic DNA of bacteriophage SPN1S (GenBank ID: JN391180.1) using primers F_LysSPN1S and R_LysSPN1S in the polymerase chain reaction. The Lys1S-L9P gene was constructed by overlapping the genes that encode KL-L9P residues (KLLKLLKKPLKLLK) at the C-terminal side of the LysSPN1S gene. In brief, the amplified native endolysin DNA sequence was re-amplified with the KL-L9P genes, F_KL-L9P(SPN1S_hm), for 12 cycles. Subsequently, the overlap-primer sets, F_overlap and R_SPN1S_L9P(N-his), were added and amplified for 18 cycles to overlap the two genes. The amplified native and engineered PCR products were digested by the BamHI and SalI restriction enzyme to include an N-terminal hexa histidine (His)-tag sequence and was ligated into the pET-28α (Novagen, Madison, WI) plasmid. The constructed recombinant plasmids were transformed into the *E. coli* BL21(DE3) strain and selected on LB agar containing 50 μg/mL kanamycin. Plasmids and primers used in this study are shown in Supplementary Tables S1, S2, respectively.

### Protein expression and purification

Constructed plasmids were transformed into *E. coli* BL21 (DE3) cells. BL21(DE3) was grown exponentially to reach an OD_600_ of approximately 0.6, induced with 0.5 mM IPTG, and incubated at 18°C with 180 rpm for 18 h for overexpression. Bacterial cells were harvested by centrifugation at 10,000 × g for 5 min and resuspended in the lysis buffer (20 mM HEPES buffer, 300 mM NaCl, pH 7.4) to be disrupted by sonication (Branson Ultrasonics, Danbury, CT). Lysates were solubilized from the membrane fraction with 2% Tween-80 at 4°C for 2 h. The supernatant was attained by centrifugation at 16,000 x g for 20 min and was passed through a Ni-NTA Superflow column (Qiagen). The resin was washed twice with washing buffer 1 (20 mM HEPES buffer, 300 mM NaCl, 1% Tween-80, 20 mM imidazole) and washing buffer 2 (20 mM HEPES buffer, 300 mM NaCl, 0.002% Tween-80, 40 mM imidazole). Endolysins were then eluted using the elution buffer (20 mM HEPES pH 7.4, 300 mM NaCl, 0.002% Tween-80, 300 mM imidazole). The elution buffer was exchanged to the storage buffer (20 mM HEPES buffer, 150 mM NaCl, 0.002% Tween-80) using PD MidiTrap G-25 (GE Healthcare) and was stored at −20°C. 15% sodium dodecyl sulfate polyacrylamide gel electrophoresis (SDS-PAGE) was used to confirm purity and sizes of the extracted endolysins. Protein concentration was estimated using the Pierce™ BCA Protein Assay Kit (Thermo Scientific).

### Checkerboard assay

The checkerboard assay was performed with minor modifications as previously described (Chang et al., 2017). LysSPN1S and KL-L9P were each serially diluted by 2-folds and placed on a 96-well plate. KL-L9P peptide was purchased from CSBio Co. (Menlo Park, United States). Bacterial suspensions of *E. coli* ATCC 25922, *A. baumannii* NCCP 15991, and *P. aeruginosa* ATCC 15692 were each added to the 96-well plate at 5 × 10^5^ CFU/mL and the plate was incubated at 37°C for 18 h. After incubation, bacterial growth (OD_600_) was measured with a SpectraMax i3 Plus Microplate Spectrophotometer (Molecular Devices, USA). The mean reduction percentage of each treatment from three biological replicates was calculated as follows: Reduction (%) = [(OD_control_ − OD_treatment_)/OD_control_] × 100. Heatmaps were produced using Gitools.

### Bactericidal activity assay

Exponentially grown *E. coli* ATCC 25922 was suspended with reaction buffer (20 mM HEPES, pH 7.4) and diluted to reach 10^6^ CFU/mL. Subsequently, bacterial cells were treated with 1 μM of each peptide or endolysin and incubated at 37°C for 1 h with shaking at 220 rpm. 100 μL of the serially diluted (1:10) samples were each dotted onto LB agar plates for enumeration. Different concentrations of endolysins were each treated to test dose-dependent activity and aliquots were extracted from each sample at the indicated time points (15, 30, 45, 60, and 120 min) to determine time-kill kinetics. All tests were performed in triplicates.

To assess the antibacterial spectrum of endolysins, Gram-negative strains listed in Supplementary Table S3 were grown to the exponential phase and were treated with 1 μM of each endolysin, following incubation at 37°C for 1 h. The bacterial suspensions were serially diluted in PBS and spotted on LB agar to be incubated at 37°C overnight.

For the storage stability test, Lys1S-L9P was stored at 4°C, −20°C, and −80°C for up to two months. After 7, 14, 30, and 60 days of storage at each temperature, exponentially grown *E. coli* ATCC 25922 was treated with 1 μM of Lys1S-L9P. Enumerations of experimental groups were conducted as described above and all experiments were performed in triplicates.

### Muralytic activity

Exponentially grown *E. coli* ATCC 25922 was resuspended in 5 mL of 0.5 M EDTA for 30 min. The membrane-disrupted cells were washed three times with autoclaved distilled water. Cells were then resuspended with the reaction buffer (20 mM HEPES, pH 7.4) to an optical density (600 nm) of 0.8 ~ 0.9 in a 96-well cell culture plate (SPL). Then, 0.2 μM of each endolysin was treated for turbidity to be measured at 600 nm of wavelength for 1 h at 37°C using SpectraMax i3 Plus Microplate Spectrophotometer(Molecular Devices, USA). Turbidity reduction assays were performed in triplicate.

### Cytotoxicity assay

HeLa (human uterus epithelial cells) and HaCaT (human skin keratinocytes) cells were cultured under standard conditions in DMEM (Gibco) containing 10% FBS (Gibco) inside a 5% CO_2_ humidified incubator at 37°C. Hela cells and HaCaT cells were seeded at a density of 5 × 10^3^ cells/well and 1 × 10^4^ cells/well, respectively, in a 96-well plate containing 100 μL of culture medium and incubated for 24 h. Next, the cells were treated with 50 μL of endolysin ranging from 0 to16 μM for 24 h. The cytotoxic effects of the endolysin and the fusion endolysin on each cell was determined using a Cytotoxicity Detection KitPLUS (Roche) following the manufacturer’s instructions. After incubation with the LDH substrate for 30 min at room temperature, the absorbance was measured at a wavelength of 490 nm using a SpectraMax i3 Plus Microplate Spectrophotometer (Molecular Devices, United States). The endolysin storage buffer (20 mM HEPES buffer, 150 mM NaCl, 0.002% Tween-80) served as a negative control. All experiments were performed in three biological and three technical replicates.

### Antibiofilm activity

Biofilm reduction assay was performed using the crystal violet staining method as previously described with minor modifications (Wu et al., 2003). In brief, *E. coli* FORC81, *A. baumannii* NCCP 1915, and *P. aeruginosa* ATCC 17543 were grown overnight in TSB-glucose (0.25% D-(+)-glucose, Sigma). Then, the bacterial cells were sub-cultured in TSB-glucose in a 96-well polystyrene microplate (SPL) and incubated at 37°C for 24 h for *E. coli* and *P. aeruginosa*, and for 72 h for *A. baumannii*. After washing bacterial cells with PBS, each endolysin was treated at various concentrations (0 to 4 μM) in each well and the reaction buffer (20 mM HEPES, pH 7.4) was used as a negative control. After a 2 h incubation at 37°C, each well was washed once with PBS and stained with 1% crystal violet at RT for 20 min. Each well was then washed three times with PBS and stains were solubilized with 33% acetic acid. Sessile biofilms were quantitated as the absorbance at 570 nm (A570) by a microplate reader. The relative A570 was calculated as follows: A_570_ test (endolysins added or buffer only) / A_570_ control (buffer only). Absorbance values for wells that contained no cells were used as blanks and subtracted from each measurement. All biofilm reduction assays were repeated in three biological replicates.

### *Galleria mellonella* as an infection model

*In vivo* antibacterial efficacy of the KL-L9P was evaluated using *Galleria mellonella* bacterial infection following the procedures by Askoura et al. with some minor modifications (Askoura and Stintzi, 2017). *G. mellonella* larvae (Ecowin, Daegu, Republic of Korea) in the final instar stage were starved in darkness for 24 h before infection. *G. mellonella* larvae were injected using a 10 μL PB600-1 repeating dispenser (Hamilton, 83700). Larvae were first injected with 10^6^ CFU bacterial culture into the last left proleg. Then, 10 μL of the storage buffer (positive control) or 10 μg of Lys1S-L9P was treated via the rear right proleg into the hemocoel within 30 min. PBS was injected instead of bacteria and treated with Lys1S-L9P as the negative control. Larvae were incubated in Petri dishes (SPL, 10090) at 37°C up to 72 h and inspected survival rates 8, 24, 48, and 72 h post infection. Larvae that did not respond to physical stimuli were considered dead. Each group consisted of 10 larvae where individual experiments were repeated twice (n = 20). Lys1S-L9P survival rates were analyzed compared to the survival rates of the positive control using the Mantel-Cox survival analysis with a log-rank test using the GraphPad Prism software.

To determine the bacterial burden on *G. mellonella* of the tested strains, larvae (n = 10) were infected with MDR bacteria and treated with Lys1S-L9P as previously described. After 24 h post infection, *G. mellonella* larvae were each disinfected with 70% ethanol and homogenized in 2 mL of sterile PBS (Dulbecco’s Phosphate-Buffered Saline, GenDEPOT) using a high-speed homogenizer (TissueRuptor, Qiagen). The homogenate was serially diluted in and plated on LB agar supplemented with streptomycin (2 μg/mL) and colistin (4 μg/mL) for *E. coli* FORC81, and gentamicin (256 μg/mL) for *A. baumannii* NCCP 15989 based on (Kim et al., 2019, 2022). Bacterial loads were counted and the Lys1S-L9P treated group was analyzed based on the control group with the independent Student’s *t*-tests using GraphPad Prism software. Data are presented as the median and interquartile range.

### Statistical analysis

The Student’s *t*-tests was performed for comparative analysis between two groups. Survival curves were analyzed by the Mantel-Cox survival analysis with log-rank test. GraphPad Prism (Version 5.01; GraphPad Software, Inc., CA, United States) was used for statistical analysis.

## Results

### Membrane-sensitizing peptide KL-L9P extends antibacterial effects of LysSPN1S against gram-negative pathogens

**Figure 1:**
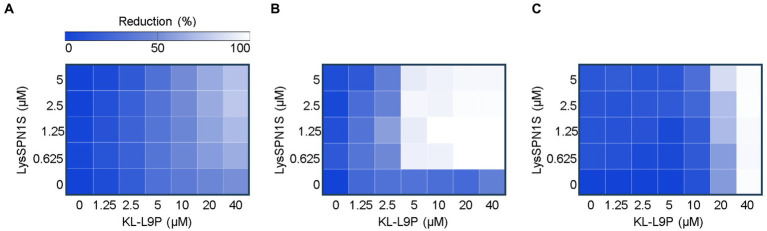
Checkerboard microdilution assay between LysSPN1S and KL-L9P. Heat maps of checkerboard assays of LysSPN1S with KL-L9P against **(A)** *E. coli*, **(B)** *A. baumannii*, and **(C)** *P. aeruginosa*. Bacterial growth was quantified by the average optical density at 600 nm (OD600). The mean reduction percentage of each treatment from three biological replicates was calculated as follows: Reduction (%) = [(ODcontrol − ODtreatment) / ODcontrol] × 100. Reduction in bacterial growth is illustrated in a linear gradient from white to blue where darker colors represent less growth inhibition.

LysSPN1S is an endolysin from the *Salmonella* phage SPN1S (GenBank ID: JN391180.1) that cleaves the glycosidic bond of peptidoglycan. LysSPN1S demonstrated remarkable capacity to induce significant bacterial reduction of membrane-permeabilized *E. coli* cells (Lim et al., 2012). The intriguing findings led to further study on this endolysin. Prior to constructing the KL-L9P peptide-fused endolysin, we performed the checkerboard assay to evaluate the potential of the sensitizer peptide KL-L9P to extend the use of endolysin to Gram-negative bacteria. The presence of KL-L9P was expected to help overcome the hydrophilic nature of LysSPN1S which hinders crossing of the Gram-negative outer membrane. Consistent with our hypothesis, the sensitizer peptide KL-L9P extended the antibacterial effects of LysSPN1S against all tested Gram-negative pathogens, *E. coli*, *A. baumannii*, and *P. aeruginosa* (Figure 1). KL-L9P peptide-independent treatments led to a gradual increase in antibacterial activity as higher concentrations was treated but required large quantities, 20 to 40 μM, to cause over a 40% reduction in all tested pathogens. Single treatments of LysSPN1S showed almost no killing effect against all tested bacteria. However, combined treatments with KL-L9P exhibited higher antibacterial effects than when treated alone. The reduction of *E. coli* and *P. aeruginosa* gradually increased with escalating doses of LysSPN1S, specifically in the presence of KL-L9P (Figures 1A,C). Moreover, treatment of 5 μM KL-L9P started to exhibit noticeably higher *A. baumannii* reduction effects even with low LysSPN1S concentrations than each of the single treatments (Figure 1B). These results support the idea that the fusion of KL-L9P to LysSPN1S could potentiate antimicrobial activities of the endolysin against Gram-negative pathogens.

### Construction of Lys1S-L9P

**Figure 2:**
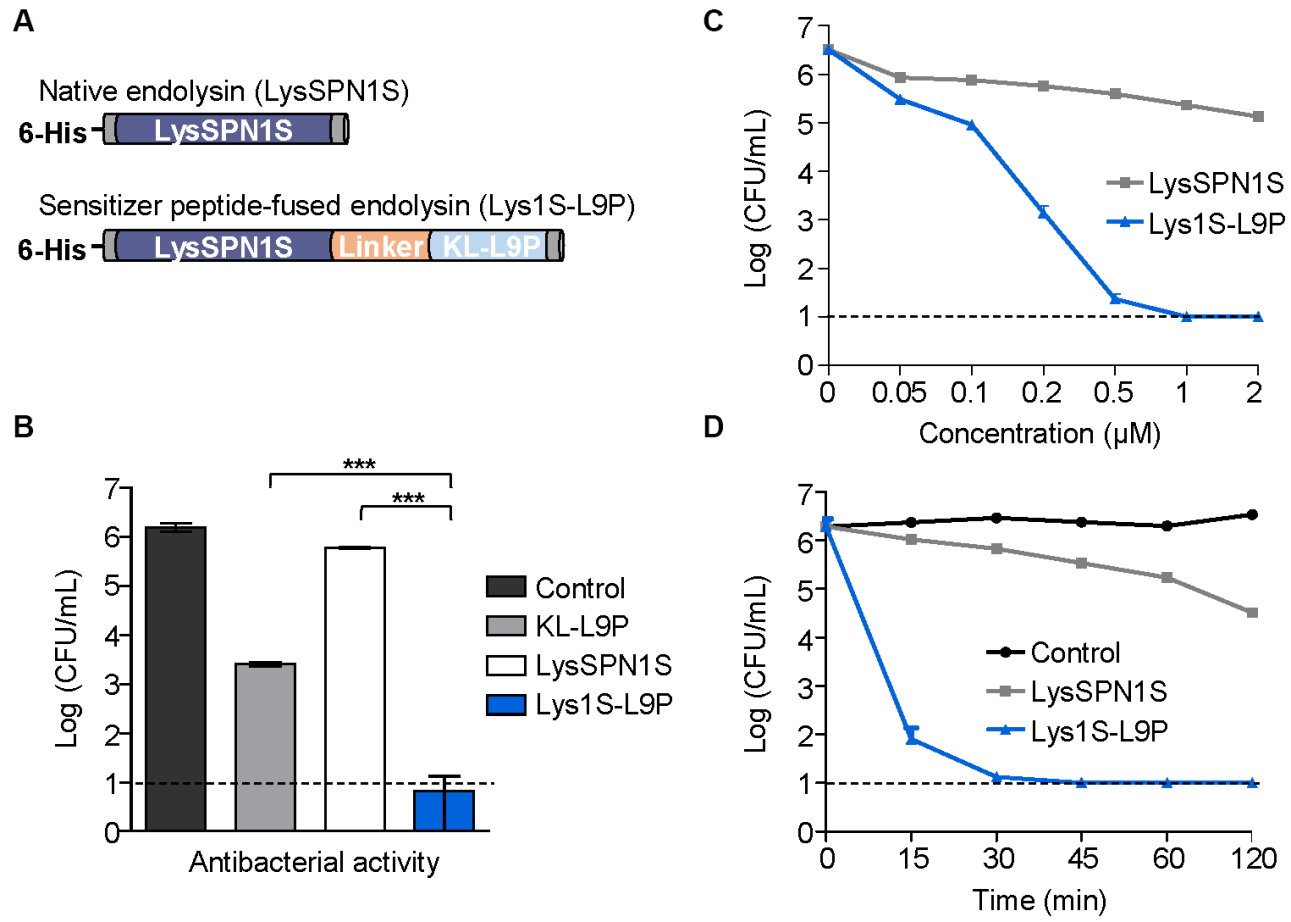
Antibacterial activity of Lys1S-L9P. **(A)** Scheme of constructing fusion endolysin. KL-L9P was fused to LysSPN1S in the C-terminal side using five amino acids (GGGGS) as a linker. **(B)** Antibacterial activity comparison of the fusion endolysin and each of the native forms. Their log-killing abilities were analyzed after 1 μM of each group was treated and incubated at 37°C for 1 h. **(C)** Dose-dependent antibacterial activity of LysSPN1S and Lys1S-L9P. Exponential phase *E. coli* was treated with each endolysin and incubated at 37°C for 1 h. **(D)** Time-kill kinetics of LysSPN1S and Lys1S-L9P. Exponential phase *E. coli* was treated with 1 μM of each endolysin and incubated at 37°C until each designated time point. All experiments were performed in triplicates. Data represent mean ± standard deviation, and the horizontal dotted lines mark the limit of detection. Statistical significance was analyzed by Student’s *t* test. ***, *p* < 0.001.

Sensitizer peptide KL-L9P was fused on the C-terminal of LysSPN1S using a linker in between (Figure 2A). The flexible linker (GGGGS) was used for its known ability to improve activity and increase stability of fusion proteins (Chen et al., 2013). The DNA sequences of the native and the engineered endolysin were each amplified, digested and ligated into pET-28α vectors to be transformed in the *E. coli* BL21(DE3) strain. Purified endolysins were confirmed using the sodium dodecyl sulfate-polyacrylamide gel electrophoresis (SDS-PAGE) analysis. The SDS-PAGE showed a single band between 25 kDa and 35 kDa bands, similar to their predicted molecular weights of LysSPN1S and Lys1S-L9P (26.7 and 28.7 kDa, respectively) confirming that the endolysins were successfully expressed and purified (Supplementary Figure 3).

### Purification of Lys1S-L9P with higher yields

The fusion endolysin resulted in low production yields, which led us towards a new approach to improve yield efficiency by utilizing the detergent, Tween-80, in the purification process. We established a new fusion endolysin purification procedure based on the solubilization method of membrane proteins with some minor modifications (Bhatt and Jeffery, 2010). Briefly, the induced bacterial cells were sonicated, and the lysate obtained from broken cells was further solubilized using 2% Tween-80. Lysates were then centrifuged, and the supernatant was purified using Ni-NTA affinity chromatography. Throughout the purification process, wash buffers were used to eliminate unwanted non-specific binding proteins, with Tween-80 concentrations in each wash buffers gradually lowered from 1% to different final concentrations. To determine the optimum Tween-80 concentration contained in the final buffer, we examined the production yield and the antibacterial activity of Lys1S-L9P, in various final buffers containing Tween-80 concentrations ranging from 0.002%, which is the critical micelle concentration (CMC) of Tween-80, to 0.02%. Results revealed that all endolysins obtained using each buffer were produced at similar protein yields of approximately 2,300 ng/μL (Supplementary Figure 1). Also, the endolysins exhibited no significant difference in the antibacterial activities against *E. coli* and *S. typhimurium*, indicating that Tween-80 in the final buffer did not affect the antibacterial activities of the Lys1S-L9P (Supplementary Figure 2). Therefore, to minimize possible cytotoxicity effects of Tween-80, we selected the buffer containing 0.002% Tween-80 as the final buffer. Surprisingly, the application of the modified procedure and the addition of Tween-80 in the lysis buffer allowed an overall 4-fold increase in the production yield of Lys1S-L9P (Supplementary Figure 3). These results suggested that our newly established purification procedure could efficiently produce peptide-fused endolysins at higher yields, which were initially difficult to purify due to their low solubility.

### Enhanced antibacterial activities of LysSPN1S fused with KL-L9P

Antibacterial activities of LysSPN1S and Lys1S-L9P were compared against a Gram-negative *E. coli* strain. One μM of KL-L9P exhibited antibacterial effects against *E. coli* causing a 2.7-log reduction while the same concentration of the native endolysin LysSPN1S caused reductions of only 0.4 logs (Figure 2B). Remarkably, 1 μM of Lys1S-L9P almost completely killed *E. coli*, reducing cells by 5.3 logs, which showed its superiority to each of the native forms. Lys1S-L9P also showed dose-dependent antibacterial activity, surpassing the effectiveness of the native endolysin. Notably, while 2 μM of the native endolysin resulted in a reduction of 1.3 logs, 0.2 μM of the fusion endolysin caused reductions of 3.3 logs and further reduced cells to under limit of detection with treatments of only 1 μM (Figure 2C). Also, the time-kill kinetics results showed that 1 μM of Lys1S-L9P was able to rapidly cause over 4-log reductions within 15 min and entirely eradicated cells to undetectable levels after 30 min (Figure 2D).

**Figure 3:**
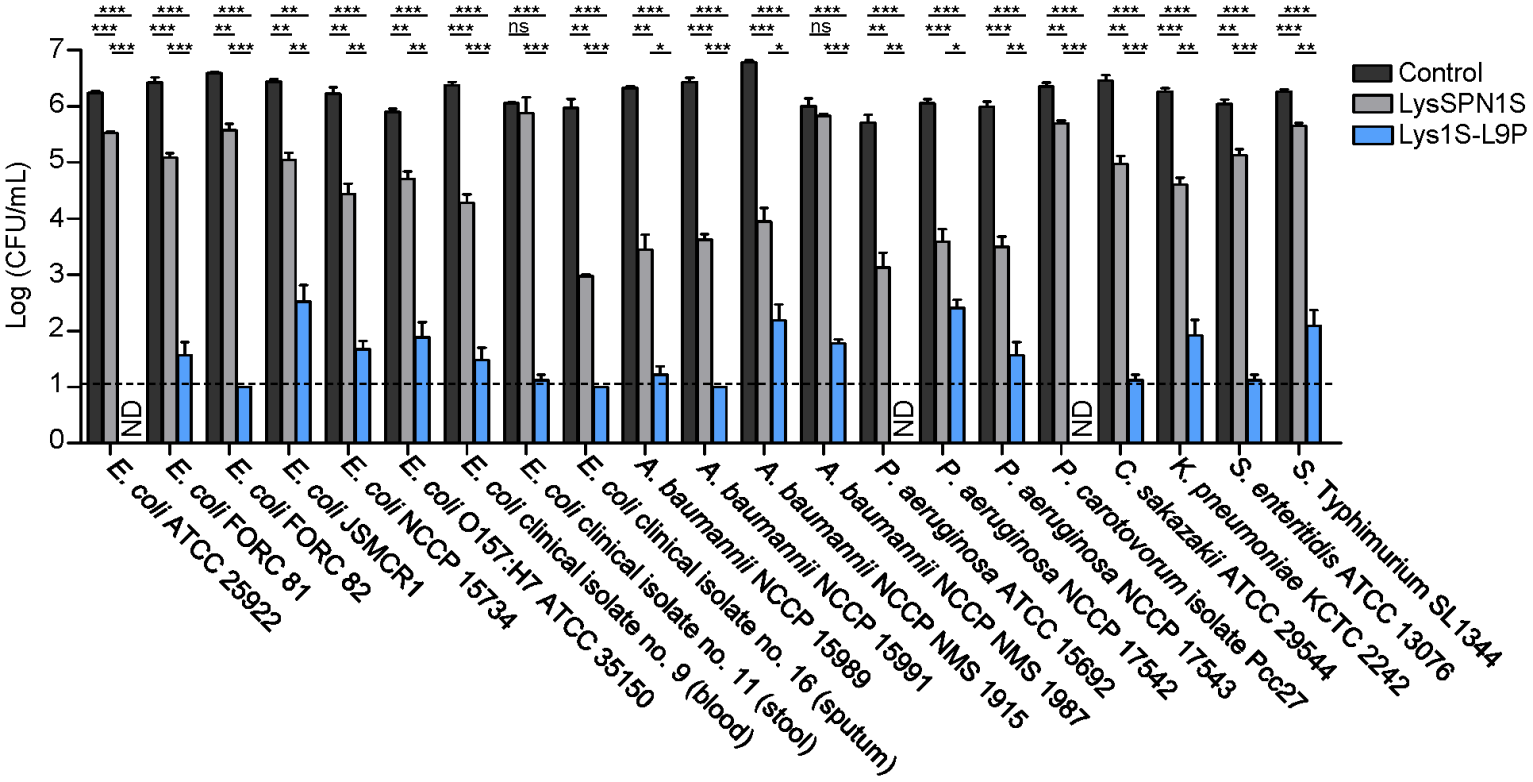
Antibacterial spectrum of LysSPN1S and Lys1S-L9P against various Gram-negative pathogens. One μM of each endolysin was incubated with each log phase bacteria at 37°C for 1 h. Experiments were performed in triplicates. Data represent mean ± standard deviation and the horizontal dotted line marks the limit of detection. Statistical significance was analyzed by Student’s *t* test. *, *p* < 0.05; **, *p* < 0.01; ***, *p* < 0.001; ns, not significant; ND, not detected.

The antimicrobial spectrum of Lys1S-L9P and the native endolysin was assessed against various Gram-negative bacteria, including several MDR strains. Strains used in this study and their antibiotic resistance information are listed in Supplementary Table S3. Lys1S-L9P exhibited a broader host spectrum compared to that of LysSPN1S and strikingly stronger lytic activity as well. All Gram-negative strains tested were highly susceptible to Lys1S-L9P, requiring only 1 μM to cause 4 or more log reductions (Figure 3). It is noteworthy that Lys1S-L9P showed highly enhanced lytic activity against colistin resistant *E. coli*, *A. baumannii*, and *P. aeruginosa* strains. These results showed that the fusion of the sensitizer peptide KL-L9P to the endolysin can potentiate lytic activity of LysSPN1S, constructing an efficient antimicrobial agent against MDR Gram-negative bacteria.

**Figure 4:**
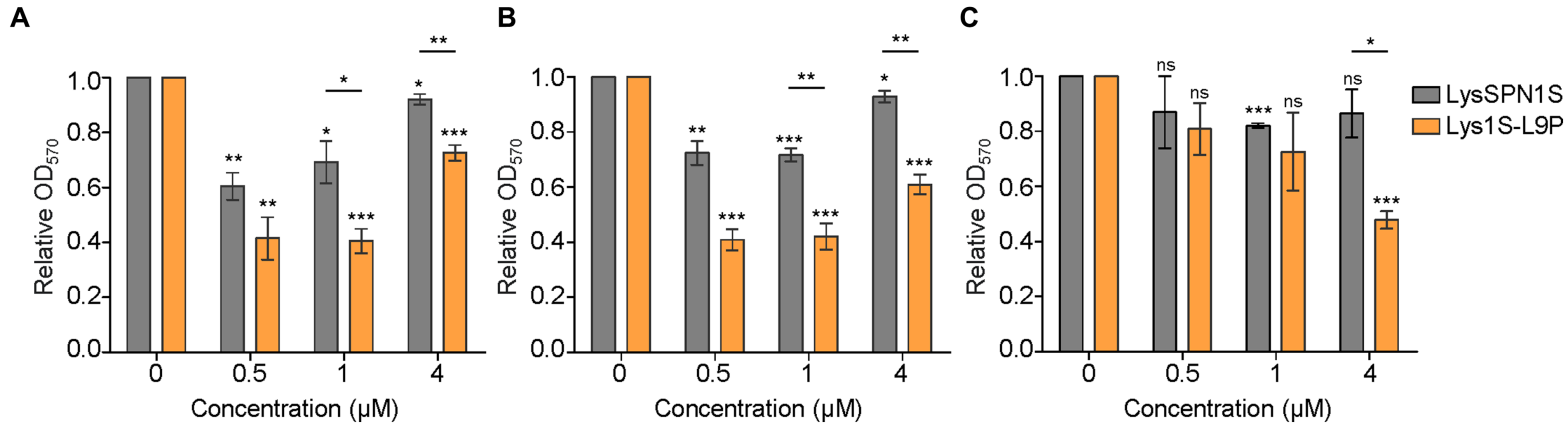
Biofilm removing efficacy of LysSPN1S and Lys1S-L9P. The biofilm removing efficacy was determined using the crystal violet staining method against three multidrug-resistant strains, **(A)** *E. coli*, **(B)** *A. baumannii*, and **(C)** *P. aeruginosa*. The error bars show the standard deviation and data were replicated for over three independent experiments. The relative A_570_ was calculated as follows: A_570_ test (endolysins added or buffer only) / A_570_ control (buffer only). Absorbance values for wells that contained no cells were used as blanks and subtracted from each measurement. Lys1S-L9P results were analyzed based on LysSPN1S results by the Student’s *t*-test and indicated above each horizontal line. Asterisks above each bar are results based on the control. *, *p* < 0.05; **, *p* < 0.01; ***, *p* < 0.001; ns, not significant.

**Antibiofilm activity.** Biofilms are sessile microbial communities that attach to surfaces and produce a matrix of extracellular polymeric substances (EPS) (Jamal et al., 2018). Biofilms may lead to an increase in antibiotic resistance up to 1,000-fold, resulting in significant challenges in clinical therapeutics (Monroe, 2007). To determine the biofilm removing efficacy of LysSPN1S and Lys1S-L9P, crystal violet (CV) staining method was performed against three MDR strains, *E. coli*, *A. baumannii*, and *P. aeruginosa*. Four μM of Lys1S-L9P showed a 52.2% decrease of OD_570_ *P. aeruginosa* whereas the same amount of LysSPN1S showed only a 13.5% reduction (Figure 4C). Lys1S-L9P was more effective against *E. coli* and *A. baumannii*, causing significant reductions in biofilm compared to the native endolysin even when only 1 μM was treated. (Figures 4A,B). The application of 1 μM of the fusion endolysin to both *E. coli* and *A. baumannii* resulted in over a two-fold decrease in biofilm with significant difference compared to the native endolysin. These results suggested that Lys1S-L9P has a more efficient biofilm removal capacity against MDR Gram-negative bacteria than the native endolysin.

### Stability and cytotoxicity

**Figure 5:**
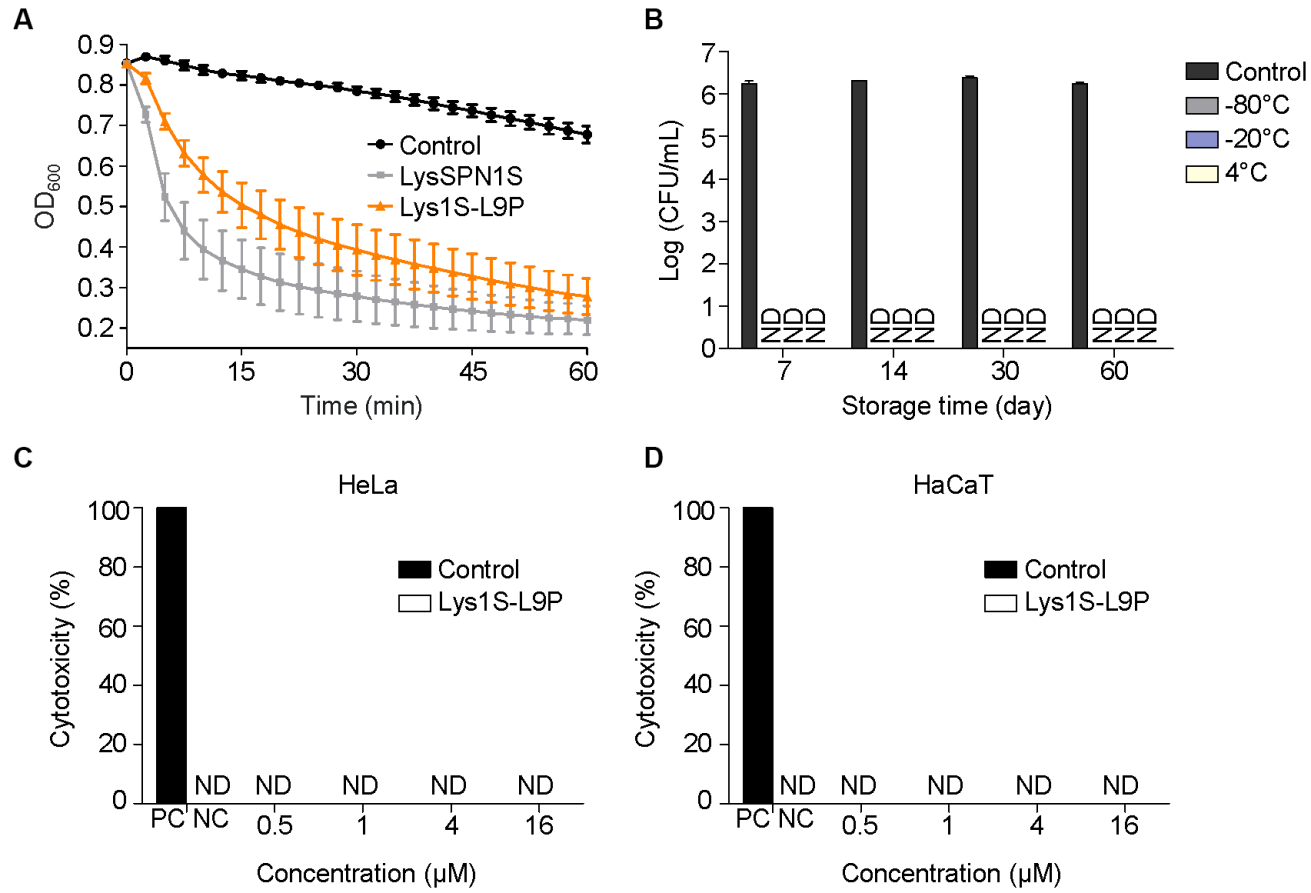
Stability and cytotoxicity of Lys1S-L9P. **(A)** Muralytic activity assay. Exponentially grown *E. coli* was treated with 0.5 M EDTA. The outer membrane permeabilized cells were treated with 0.2 μM of LysSPN1S and Lys1S-L9P in a 96 well cell culture plate. **(B)** Storage stability test of Lys1S-L9P at various temperatures. Exponential phase *E. coli* was incubated with 1 μM of Lys1S-L9P for 1 h at each designated time point. Cytotoxicity of Lys1S-L9P against **(C)** HeLa and **(D)** HaCaT cells. Cytotoxicity was measured using the LDH Cytotoxicity Detection Kit (Roche) according to the manufacturer’s instructions. Experiments were performed in triplicates and data represent mean ± standard deviation. PC, positive control; NC, negative control; ND, not detected.

To determine whether the peptidoglycan degrading ability of the native endolysin was hampered by the fusion of the sensitizer peptide, muralytic assay was performed (Figure 5A). Briefly, *E. coli* cells were treated with each endolysin after destabilizing the outer membrane of the cells with EDTA. Absorbance at 600 nm was then measured at specified time points. LysSPN1S and Lys1S-L9P both exhibited similar rates of turbidity reduction in the EDTA treated cells, which implies that the fusion of the sensitizer peptide did not affect the peptidoglycan degrading ability of the endolysin.

To be suitable for clinical use, therapeutic agents are required to ensure stability during storage and safety to the human cells. Storage stability of Lys1S-L9P at −80°C, −20°C and 4°C was evaluated against log phase *E. coli* at each day point for up to two months. Results revealed that Lys1S-L9P was stable for up to two months without any activity loss in all storage temperatures (Figure 5B). The cytotoxicity of Lys1S-L9P was evaluated using the lactate dehydrogenase (LDH) assay. Human uterus epithelial cells (Hela) and human skin keratinocytes (HaCaT) were incubated with a high dose of up to 16 μM of Lys1S-L9P for 24 h. After the catalyst LDH and dye solution were applied to each well, cell viability was quantified by measuring the absorbance at 490 nm. The results showed that the highest dose of Lys1S-L9P was less likely to exhibit cytotoxicity against both human cells (Figures 5C,D), suggesting Lys1S-L9P could be utilized as a potential safe treatment.

### *In vivo* efficiency test using the *Galleria mellonella* infection model

**Figure 6:**
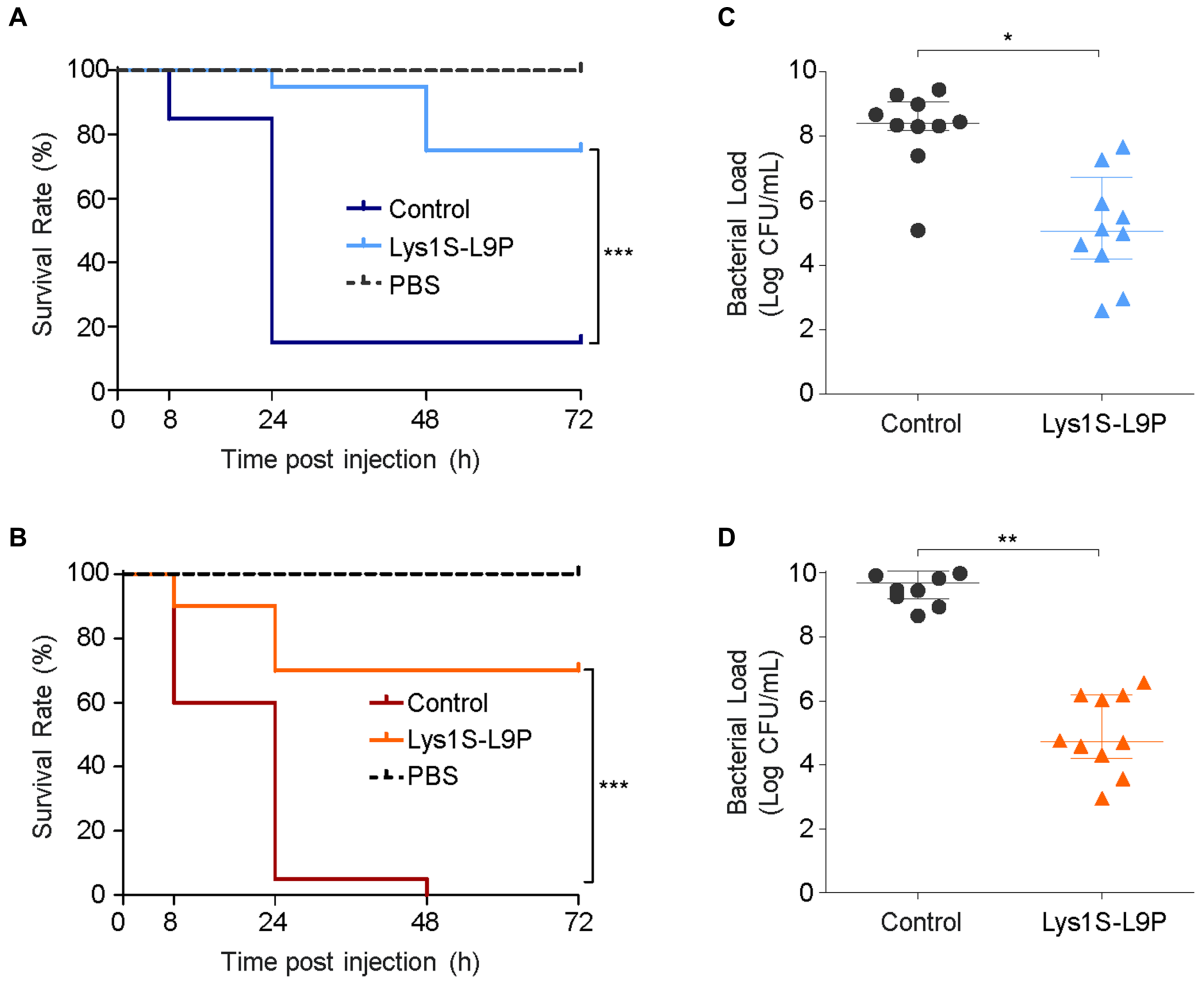
*In vivo* antibacterial efficacy of Lys1S-L9P in *Galleria mellonella*. Survival curves of *G. mellonella* larvae infected with 10^6^ CFU **(A)** *E. coli* and **(B)** *A. baumannii.* Horizontal dotted lines show negative control groups, where larvae were injected with PBS containing no bacteria and treated with Lys1S-L9P. The positive control were treated with PBS after bacterial infection. 10 μg of Lys1S-L9P was treated within 30 min post infection (*n* = 20). Data were analyzed by the Mantel-Cox survival analysis with log-rank test. Bacterial counts of **(C)** *E. coli* and **(D)** *A. baumannii* in homogenized larvae of the control group and the treatment group after 24 h (*n* = 10). Data are presented as the median and the interquartile range. Statistical significance was analyzed by Student’s *t* test. *, *p* < 0.05; **, *p* < 0.01; ***, *p* < 0.0001.

To assess the therapeutic potential of Lys1S-L9P in *in vivo* efficiency, we analyzed the survival rates and bacterial burden reduction effects using the *G. mellonella* infection model. Briefly, the left rear proleg was initially injected with 10^6^ CFU, followed by the injection of the storage buffer (control) or Lys1S-L9P into the right rear proleg within 30 min. As the negative control, phosphate-buffered saline (PBS) was injected instead of bacteria and Lys1S-L9P was treated within 30 min. The negative control group maintained survival rates of 100%, which implies that Lys1S-L9P is nontoxic towards *G. mellonella* larvae. In the group treated with MDR *E. coli*, survival rates decreased to approximately 15% within 24 h. In contrast, 95% of the group treated with Lys1S-L9P survived 24 h post inoculation and maintained survival rates to 75% even after 72 h post inoculation (Figure 6A). MDR *A. baumannii* infected larvae survival rates dropped from 60% after 8 h to 5% after 24 h, and all larvae died after 48 h of injection. On the other hand, the Lys1S-L9P treatment rescued 90% of larvae 8 h post inoculation and maintained survival rates at 70% from 24 to 72 h post injection (Figure 6B). Collectively, the survival rate in the fusion endolysin treatment group was significantly higher than the non-treated control group, which indicates that the fusion endolysin successfully rescued *G. mellonella* from bacterial infection.

For quantification of *in vivo* antibacterial efficacy, we measured the bacterial burden in *G. mellonella*. Ten larvae in each Lys1S-L9P treated or non-treated groups were homogenized at 24 h post inoculation. Homogenates were then serially diluted and plated to count bacterial loads. The bacterial counts of the enzyme-treated group showed significant reductions compared to that of the buffer control, exhibiting 2 log and 3.9 log reductions against *E. coli* and *A. baumannii*, respectively (Figures 6C,D). The significant efficacy of Lys1S-L9P in the *G. mellonella* infection model demonstrates that Lys1S-L9P could be considered as a potential treatment for highly concerning MDR bacterial infections.

## Discussion

Bacteriophage-derived lytic enzymes, endolysins, are promising alternatives to conventional antibiotics but face difficulties in controlling Gram-negative bacteria due to their additional outer membrane. Several protein engineering concepts have been devised to overcome this barrier, but problems such as low solubility, narrow host ranges, and low efficiency in the *in vivo* model still remain to be solved. Therefore, new protein engineering approaches are in demand to overcome such shortcomings, but recent research on peptide-fused endolysins is limited to certain peptides, such as cecropin A (Hong et al., 2022; Islam et al., 2022; Lim et al., 2022; Jeong et al., 2023). Sensitizer peptides can alter the structure of the outer membrane of Gram-negative bacteria without abolishing bacterial membranes. They facilitate relocation of anionic LPS molecules to positions where they can no longer prevent penetration of antimicrobial agents. Based on this background, we selected the peptide, KL-L9P, to construct a novel peptide fused endolysin that extends antibacterial effects to Gram-negative bacteria. The checkerboard assay results using KL-L9P and LysSPN1S revealed that the lytic activity of LysSPN1S was gradually potentiated as higher concentrations of KL-L9P was treated (Figure 1). Based on previous findings, the sensitizer peptide KL-L9P did not create holes in the membrane, rarely damaging outer or inner membranes of *E. coli* (Hyun et al., 2020). It is therefore feasible to say that KL-L9P could more likely have acted as an adjuvant to the endolysin against impermeable cell walls of Gram-negative bacteria and allowed the endolysin to cross the outer membrane barrier, rather than showing synergy with LysSPN1S. These results supported the validity of the idea that the fusion of sensitizer peptides to the endolysin could work as efficient potential antimicrobial agents active against MDR Gram-negative bacteria.

The KL-L9P sensitizer peptide was fused on to the LysSPN1S endolysin, developing a novel fusion endolysin, Lys1S-L9P. However, the fusion endolysin resulted in low production yields. This could be because the hydrophobicity of the peptide can promote interaction with neighboring proteins or increase the likelihood of insertion into cell membranes. Therefore, we aimed to reduce any of these possible effects and improve yield efficiency by introducing the use of detergent in the purification process. We compared the solubilization effect of two FDA-approved detergents, Tween-20 and Tween-80, and confirmed that only Tween-80 increased production yields of Lys1S-L9P (data not shown). Several final concentrations of Tween-80 were examined and there was no significant difference in production yields or antibacterial activity. Therefore, the CMC of Tween-80 was selected to be contained in the final buffer to minimize potential toxicity. This modified purification process allowed a 4-fold increase of production yield in Lys1S-L9P (Supplementary Figure 3). Our newly established purification method can be applied in other future peptide-fused endolysins to reach higher production yields.

The fusion endolysin Lys1S-L9P exhibited remarkable potential as a therapeutic agent for MDR Gram-negative bacteria. The fusion of the sensitizer peptide significantly enhanced antimicrobial activity compared to each native forms, LysSPN1S and KL-L9P, with no hindrance in its native muralytic activity (Figures 2B, 5A). Also, Lys1S-L9P showed significantly higher lytic activity among a broader spectrum of MDR Gram-negative bacteria (Figure 3). Surprisingly, Lys1S-L9P efficiently killed colistin-resistant *E. coli* with only a treatment of 1 μM (Figure 3). Colistin is known to displace divalent cations in a competitive manner, self-promoting itself through the outer membrane, ultimately causing cell lysis (Velkov et al., 2010; Andrade et al., 2020). Unlike colistin, KL-L9P does not demolish but promotes rearrangement of the outer membrane (Hyun et al., 2020). The difference between colistin and KL-L9P in their interaction with the outer membrane of Gram-negative bacteria might be the reason as to why Lys1S-L9P was able to kill colistin-resistant strains. In addition, Lys1S-L9P exhibited higher efficacy in biofilm eradication of *E. coli*, *A. baumannii*, and *P. aeruginosa* compared to the native endolysin LysSPN1S (Figure 4). Interestingly, high concentrations of LysSPN1S and Lys1S-L9P exhibited less efficiency in *E. coli* and *A. baumannii* biofilm reduction. This can possibly be explained as the result of competitive inhibition between binding and cutting sites of the endolysin in bacterial surface occupation due to high concentrations in static conditions (Kuiper et al., 2021).

Lys1S-L9P also satisfies conditions for clinical use, with high potential in safety and stability. *E. coli* was selected for storage stability assessment because it is a well-known representative of Gram-negative bacteria and its cell wall structure is similar to most of the other Gram-negative bacteria which have a highly conserved A1 γ-type peptidoglycan structure. The storage stability of Lys1S-L9P was prolonged, maintaining its initial lytic activity against *E. coli* even after storage at −80°C, −20°C, 4°C for 2 months (Figure 5B). This is a notable difference compared to some endolysins that started to lose stability after 1 month of storage at 4°C (Maciejewska et al., 2017; Chen et al., 2021). Cytotoxicity assays revealed that Lys1S-L9P rarely exhibited cytotoxicity against HeLa and HaCaT cells even at high concentrations of Lys1S-L9P (Figures 5C,D). These results implied that Lys1S-L9P could have high potential as an efficient therapeutic agent against various MDR Gram-negative bacteria, including those resistant to the last resort antibiotic, colistin.

The *G. mellonella* infection model is the simplest and cheapest way to conduct initial *in vivo* tests of new antibacterial compounds before being testing on mammals (Desbois and Coote, 2012). It is mostly free from ethical concerns compared to murine models (Wojda et al., 2020). Moreover, their innate immune response has similarity to mammals and correlates with murine immunity (Brennan et al., 2002; Slater et al., 2011). Survival of infected *G. mellonella* larvae was significantly higher when Lys1S-L9P was treated compared to the control. Survival rates after the treatment of Lys1S-L9P against *E. coli* and *A. baumannii* infected larvae were 75% and 70%, respectively, and bacterial loads of infected larvae were significantly reduced (Figure 6). The encouraging results in the *in vivo G. mellonella* infection model revealed great potential of Lys1S-L9P to be utilized as a therapeutic agent. Collectively, this study established a foundation for subsequent preclinical assessment of Lys1S-L9P, paving the way for further potential applications and investigations.

## Conclusion

In the present work, we developed a novel fusion endolysin, Lys1S-L9P, using the sensitizer peptide KL-L9P and established a strategy for high-yield productions of the peptide-fused endolysin. The fusion endolysin had a stronger and broader host range against MDR Gram-negative strains, including those resistant to colistin. The prolonged storage, low cytotoxicity, high antibiofilm activity, and efficiency in *in vivo* tests proved Lys1S-L9P to be a great potential therapeutic agent. Our findings demonstrated the strong potential of utilizing sensitizer peptides in endolysin engineering and showed promising potential of Lys1S-L9P as a therapeutic agent to combat MDR Gram-negative bacteria.

## Data availability statement

The dataset presented in this study can be found in online repositories. The name of the repository and accession number can be found below: NCBI: JN391180.1 (ncbi.nlm.nih.gov/nuccore/JN391180.1).

## Author contributions

SS: Investigation, Methodology, Writing – original draft. JK: Investigation, Methodology, Writing – original draft. SR: Conceptualization, Funding acquisition, Supervision, Writing – review & editing.
