# Supplementary material for: Development of sensitizer peptide-fused endolysin Lys1S-L9P acting against multidrug-resistant gram-negative bacteria
Source: Front Microbiol. 2023 Nov 23;14:1296796. doi: 10.3389/fmicb.2023.1296796 (PMC10701683; doi:10.3389/fmicb.2023.1296796)
Supplement: Supplementary file 1 [file Data_Sheet_1.docx]

Supplementary Material


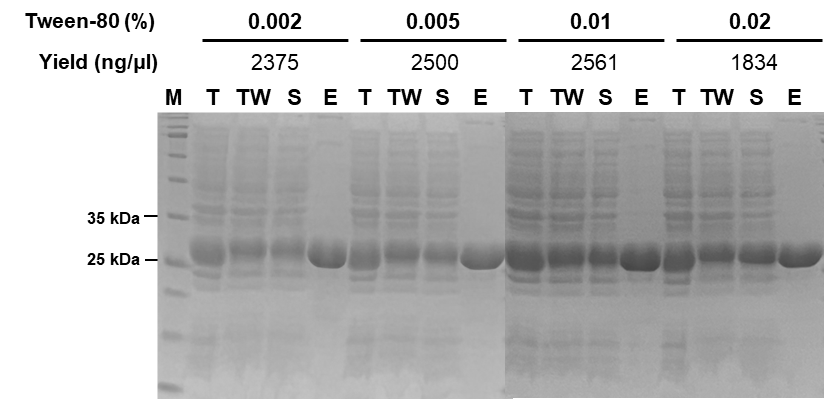


**Supplementary Figure 1. SDS-PAGE analysis of Lys1S-L9P in different Tween-80 concentrations.** Induced expressions of Lys1S-L9P (28.7 kDa) were analyzed on 15% SDS-PAGE gel. Tween-80 concentrations contained in the final buffer are noted above the bar, and the overall production yield is noted below the bar. Lane Legends: M, marker; T, total lysate; TW, with 2% Tween-80; S, soluble lysate; E, elution.


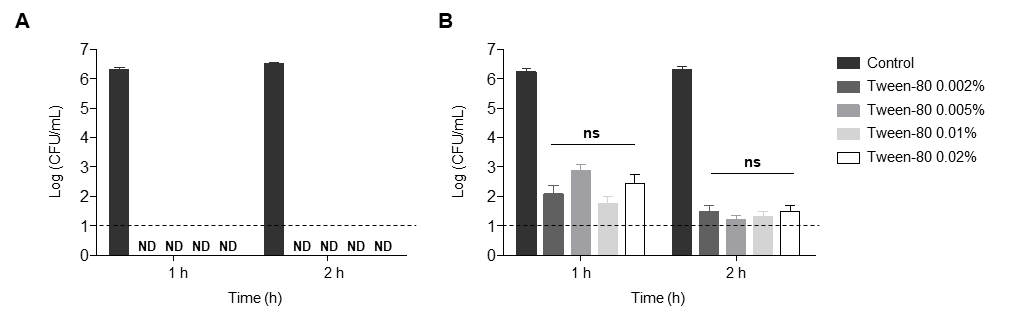


**Supplementary Figure 2. Antibacterial activity of Lys1S-L9P stored in different Tween-80 concentrations.** The log killing assay was conducted against (A) *E. coli* ATCC 25922 and (B) *S.* Typhimurium SL1344. Each final buffer containing different Tween-80 concentrations is indicated in each legend. 1 μM of each endolysin was treated against exponentially grown bacteria and incubated at 37°C until the designated time points. Experiments were performed in triplicates. Data represent mean ± standard deviation and horizontal black lines mark the limit of detection. Statistical significance was analyzed by Student’s *t*-test. ND, not detected; ns, not significant.


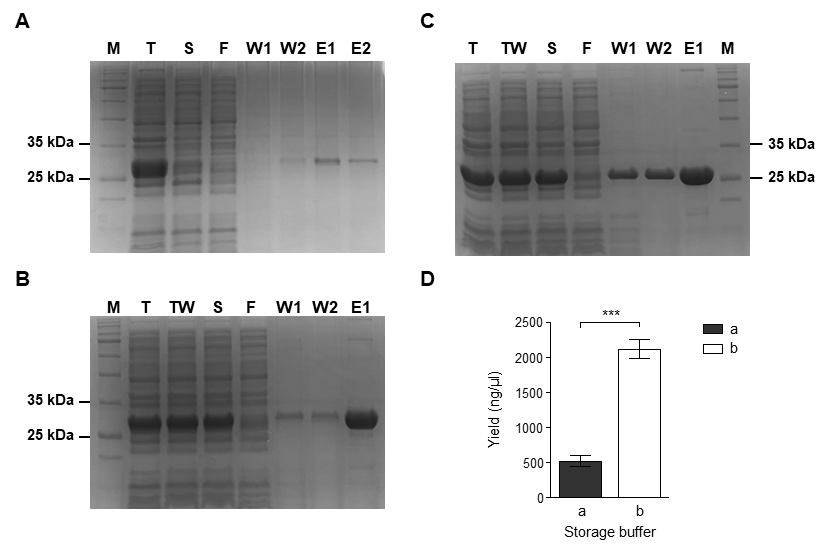


**Supplementary Figure 3. Production yield of Lys1S-L9P.** SDS-PAGE analysis of Lys1S-L9P (28.7 kDa) obtained by (A) standard purification methods and by (B) our modified purification process. (C) SDS-PAGE analysis of LysSPN1S (26.7 kDa) in the final buffer containing 0.002% Tween80. (D) Lys1S-L9P production yield comparison. Legend a represents buffer consisting of 20 mM HEPES and 150 mM NaCl. Legend b represents the final buffer (20 mM HEPES, 150 mM NaCl, 0.002% Tween-80). Error bars indicate standard deviation and data were replicated for three independent experiments. Statistical significance was analyzed by Student’s *t*-test. ***, *P* < 0.001. Lane Legends: M, marker; T, total lysate; TW, with Tween-80; S, soluble lysate; F, flow through after Ni-His tag binding; W1, washing with 20 mM imidazole; W2, washing with 40 mM imidazole; E1, elution with 300 mM imidazole; E2, elution with 300 mM imidazole.

**Supplementary Table 1. Primers used in this study**

| **Primer** | **Sequence (5`- 3`)** |
| --- | --- |
| F_LysSPN1S | AAA GGA TCC ATG GAC ATT AAC CAG TTC CGG C |
| R_LysSPN1S | GCT GCC GCC GCC GCC TAA CGC CAG CAC CTT ACG GG |
| F_KL-L9P(SPN1S_hm) | GCT GGC GTT AGG CGG CGG CGG CAG CAA ACT GCT GAA ACT GCT GAA GAA ACC GCT GAA ACT GCT GAA ATG A |
| F_overlap | GGA TCC ATG GAC ATT AAC CAG TT |
| R_SPN1S_L9P(N-his) | AAA GTC GAC TCA TTT CAG CAG TTT CAG CGG TT |
| R_SPN1S_(N-his) | AAA GTC GAC TCA TAA CGC CAG CAC CTT ACG GGC A |

**Supplementary Table 2. Plasmids used in this study**

| **Plasmids** | **Characteristics** |
| --- | --- |
| pET28a::LysSPN1S_(N) | Kan^R^, T7 Promoter, IPTG induced, hexa his-tagged |
| pET28a::Lys1S-L9P_(N) | Kan^R^, T7 Promoter, IPTG induced,  N-terminal LysSPN1S, C-terminal KL-L9P, hexa his-tagged |

**Supplementary Table 3. Strains used in this study**

| **Strain^a^** | **Antibiotic resistance^b^** | **Reference or Source^c^** |
| --- | --- | --- |
| **- *E. coli* strains** |  |  |
| *Escherichia coli* ATCC 25922 | Unknown | ATCC |
| *Escherichia coli* FORC 81 | AMP, CEF, CHL, CIP, COL, GEN, KAN, POL, STR, TET | (Kim et al., 2019) |
| *Escherichia coli* FORC 82 | AMP, CEF, CHL, COL, POL,TET | (Kim et al., 2019) |
| *Escherichia coli* JSMCR1 | AMP, CEF, CHL, CIP, COL, GEN, KAN, POL, STR, TET | (Kim et al., 2019) |
| *Escherichia coli* NCCP 15734 | AMC, AMP, FEP, FOX, GEN, NAL | NCCP |
| *Escherichia coli* O157:H7 ATCC 35150 | Unknown | ATCC |
| *Escherichia coli* clinical isolate no. 9 | ATM, CAZ, CIP, FEP, IPM, MEM, PIP, TZP | Blood |
| *Escherichia coli* clinical isolate no. 11 | AN, ATM, CAZ, CIP, FEP, GEN, IPM, MEM, PIP, TZP | Stool |
| *Escherichia coli* clinical isolate no. 16 | ATM, CAZ, CIP, FEP, IPM, MEM, PIP, TZP | Sputum |
| **- Other Gram-negative bacteria** |  |  |
| *Acinetobacter baumannii* NCCP 15989 | CC, OXA, ST, MEM, DOX, GEN, ERY | NCCP,  (Kim et al., 2022) |
| *Acinetobacter baumannii* NCCP 15991 | CC, OXA, ST | NCCP |
| *Acinetobacter baumannii* NCCP 1915 | CIP, COL, MEM | NCCP |
| *Acinetobacter baumannii* NCCP 1987 | Unknown | NCCP |
| *Pseudomonas aeruginosa* ATCC 15692 | Unknown | ATCC |
| *Pseudomonas aeruginosa* NCCP 17542 | AN, ATM, CAZ, CIP, COL, DOR, FDC, FEP,  GEN, I-R, IPM, LVX, MEM, TOB, TZP | NCCP |
| *Pseudomonas aeruginosa* NCCP 17543 | CAZ, CIP, COL, DOR, FEP, GEN, IPM, LVX, MEM, TOB, TZP | NCCP |
| *Pectobacterium carotovorum* isolate Pcc27 | Unknown | - |
| *Cronobacter sakazakii* ATCC 29544 | Unknown | ATCC |
| *Klebsiella pneumoniae* KCTC 2242 | Unknown | KCTC |
| *Salmonella enteritidis* ATCC 13076 | Unknown | ATCC |
| *Salmonella* Typhimurium SL1344 | Unknown | ATCC |

^a^ATCC, American Type Culture Collection; KCTC, Korean Collection for Type Cultures; NCCP, National Culture Collection for Pathogens.

^b^AMC, Amoxicillin; AMP, Ampicillin; AN, Amikacin; ATM, Aztreonam; CAZ, Ceftazidime; CC, Clindamycin; CIP, Ciprofloxacin; COL, Colistin; DOR, Doripenem; DOX, Doxycycline; ERY, Erythromycin; FDC, Cefiderocol; FEP, Cefepime; FOX, Cefoxitin; GEN, Gentamicin; I-R, Imipenem-relebactam; IPM, Imipenem; LVX, Levofloxacin; MEM, Meropenem; NAL, Nalidixic acid; OXA, Oxacillin; PIP, Piperacillin; ST, Streptomycin; SXT, Trimethoprim; TET, Tetracycline; TOB, Tobramycin; TZP, Piperacillin + Tazobactam.

**^c^**-, not available

**REFERENCE**

Kim J, Hwang BK, Choi H, Wang Y, Choi SH, Ryu S, et al. Characterization of mcr-1-Harboring Plasmids from Pan Drug-Resistant *Escherichia coli* Strains Isolated from Retail Raw Chicken in South Korea. Microorganisms. 2019;7:344. https://doi.org/10.3390/microorganisms7100470.

Kim SH, Yun S, Park W. Constitutive Phenotypic Modification of Lipid A in Clinical *Acinetobacter baumannii* Isolates. Microbiol Spectr. 2022;10:e0129522. https://doi.org/10.1128/spectrum.01295-22.
